# Periostin-integrin signaling in hepatocellular carcinoma: from biological function to clinical application

**DOI:** 10.3389/fcell.2025.1520739

**Published:** 2025-09-12

**Authors:** Jun Lei, Yong Liu, Shuai Yuan, Xiaxia Yuan, Qi Yuan

**Affiliations:** ^1^ Department of State-owned Assets Management, Mudanjiang Medical University, Mudanjiang, China; ^2^ Center for Comparative Medicine, Mudanjiang Medical University, Mudanjiang, China; ^3^ School of Basic Medicine, Qiqihar Medical University, Qiqihar, China; ^4^ Department of Life Science and Engineering, Jining University, Qufu, China; ^5^ College of Life Sciences, Mudanjiang Medical University, Mudanjiang, China

**Keywords:** hepatocellular carcinoma, periostin, integrin, extracellular matrix, cancer-associated fibroblasts

## Abstract

Hepatocellular carcinoma (HCC) is the third leading cause of cancer death worldwide and the most common primary tumor. Periostin (POSTN) is located in the extracellular matrix (ECM) and triggers tumor growth signals by binding to integrin receptors. The interaction of highly expressed POSTN with cell surface receptor integrins regulates intracellular signaling pathways and promotes HCC progression. In this review, the structure and isoforms of POSTN will be summarized, and the relationship between POSTN-integrin signaling and the diagnosis and prognosis of HCC patients, tumor cell proliferation and metastasis, immune escape, cancer stem cells and angiogenesis will be reviewed. The interaction between POSTN-integrin and the key signaling pathways of HCC and its mechanism in disease progression were emphasized, and the potential value of this signaling axis as a therapeutic target for HCC was explored, providing a theoretical basis for in-depth understanding of the pathophysiological process of HCC and the development of new therapeutic strategies.

## 1 Introduction

Hepatocellular carcinoma (HCC) represents a significant global health challenge, being the most common form of liver cancer and a leading cause of cancer-related mortality (Singal et al., 2023; Yang et al., 2022). The rising incidence of HCC is primarily attributed to risk factors such as chronic viral hepatitis, alcohol consumption, liver fibrosis and metabolic syndrome (Singal et al., 2023; Toh et al., 2023; Shi et al., 2023). Despite advances in diagnostic techniques and therapeutic approaches, the 5-year survival rate of HCC patients after clinical surgery and adjuvant drug therapy exceeds 30% (Singal et al., 2023), postoperative recurrence and metastasis are still the major threats to poor prognosis of HCC patients. The complex molecular mechanisms that drive HCC relapse and confer systemic therapy resistance remain poorly understood, highlighting the urgent need for a deeper understanding of the underlying molecular mechanisms that drive tumor development and progression.

Recent research has highlighted the importance of the tumor microenvironment (TME) in cancer development, where interactions between tumor cells and surrounding stromal components play a pivotal role in modulating tumor behavior (Kennel et al., 2023; Donne and Lujambio, 2023; Cheng et al., 2022). Among the various extracellular matrix (ECM) proteins involved, periostin (POSTN) has emerged as a key player in several malignancies (Dorafshan et al., 2022; Nikoloudaki et al., 2020; Nie et al., 2020; Bergmann et al., 2022). POSTN is produced in response to tissue injury and inflammation, often leading to its upregulation in various solid tumors, including HCC, esophageal cancer, colorectal cancer and lung cancer (Xiao et al., 2021; Chen K. et al., 2020; Ueki et al., 2023; Ratajczak-Wielgomas et al., 2022). This matricellular protein is known for its ability to interact with integrin receptors on the cell surface, initiating signaling cascades that influence cell adhesion, migration and survival.

The POSTN-integrin signaling axis has garnered attention due to its involvement in several processes critical for HCC progression, including epithelial-mesenchymal transition (EMT), angiogenesis, and the establishment of a pro-tumorigenic microenvironment (Xiao et al., 2021; Chen B. C. et al., 2020; Chen et al., 2017). These processes not only facilitate tumor growth and metastasis but also contribute to resistance against conventional therapies. Given the complex interplay between POSTN-integrin signaling and HCC, understanding this signaling pathway may reveal novel therapeutic targets and strategies.

This review aims to provide a comprehensive overview of POSTN-integrin signaling in the context of HCC. We will explore the structural and functional characteristics of POSTN, elucidate the mechanisms through which it engages integrin receptors, and discuss its role in HCC pathophysiology. Additionally, we will examine the potential therapeutic implications of targeting POSTN-integrin signaling as a novel approach to improving outcomes for HCC patients. We seek to contribute to the growing body of knowledge surrounding POSTN-integrin signaling and its implications for HCC patients, ultimately contributing to the development of innovative strategies for diagnosis and treatment in this challenging disease.

## 2 Molecular properties of POSTN and integrin

### 2.1 POSTN structure

POSTN, also known as osteoblast-specific factor 2 (OSF-2), is a 90-kDa secreted extracellular matrix protein belonging to the fasciclin family, which is widely distributed in collagen-rich connective tissues (Takeshita et al., 1993; Coutu et al., 2008; Pickering et al., 2023). Sequence analysis has shown that the homology of POSTN between human and mouse is 89.2% (Takeshita et al., 1993). In mice, it is located on chromosome 3C and has 25 exons. In humans, however, there are 24 exons on the long arm of chromosome 13 (13q13.3) (Dorafshan et al., 2022; González-González and Alonso, 2018). The terminal exons of both mice and human are protein-coding regions, encoding proteins of 836 and 838 amino acids, respectively (Dorafshan et al., 2022; Yu et al., 2021).

**FIGURE 1:**
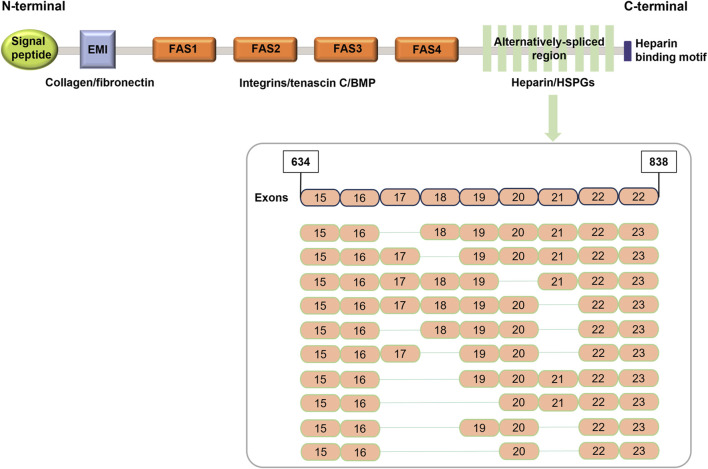
Schematic representation of POSTN structure (modified from Dorafshan et al. (2022)).

POSTN consists of a signal peptide secreted from the N-terminal, a cysteine-rich domain (EMI domain), four internal homologous repeat domains (FAS domain), and a selectively spliced C-terminal hydrophilic domain (Wang et al., 2022; Sonnenberg-Riethmacher et al., 2021; Yoshihara et al., 2023) (Figure 1). In the EMI domain, collagen and fibronectin interact. However, in the FAS domain, integrin, tendinin C, bone morphogenetic protein 1 (BMP-1), and cell communication network factor 3 (CCN3) bind to each other (Takayama et al., 2017; Kii et al., 2010; Hwang et al., 2014). The C-terminal domain binds to heparin and heparin sulfate proteoglycans (HSPGs) and can undergo selective splicing (Kii and Ito, 2017).

### 2.2 Integrin

Integrins containing extracellular, transmembrane and cytoplasmic domains exist on the cell surface. Integrins mediate recognition between cells and extracellular matrix, depending on Ca^2+^ or Mg^2+^ adhesion molecules (Kechagia et al., 2019). To date, 18 α and 8 β subunits have been reported to constitute 24 different heterodimer integrin receptors (Chen et al., 2021). The α-subunit determines ligand specificity, while the β-subunit connects the cytoskeleton and influences signaling pathways (Hynes, 2002; Campbell and Humphries, 2011). Among these integrins, α_v_β_3_, α_v_β_5_, α_6_β_4_ and α_m_β_2_ have been shown to function as POSTN receptors (Guarino, 2007; De Oliveira Macena et al., 2024; Vimalatha et al., 2018; Bao et al., 2004).

## 3 POSTN and integrin expression in HCC

POSTN is expressed in various tissues, including bone, skin, and the heart, but its expression is markedly elevated in pathological conditions, particularly in cancer (Dorafshan et al., 2022; Murota et al., 2017; Valiente-Alandi et al., 2016). The expression level of POSTN is often upregulated and is associated with tumor aggressiveness and poor prognosis in HCC patients (Wang et al., 2024a; Li Z. et al., 2024). Factors such as chronic inflammation, hypoxia, and the activation of signaling pathways (e.g., transforming growth factor β (TGF-β) and interleukin 6 (IL-6)) contribute to POSTN upregulation. POSTN is frequently overexpressed in the HCC TME. It is secreted primarily by cancer-associated fibroblasts (CAFs), activated hepatic stellate cells (HSCs), and, to a lesser extent, tumor cells themselves (Chen et al., 2021; Wang et al., 2024a; Zhang R. et al., 2018). The main function of POSTN in the ECM is to interact with integrins, particularly α_v_β_3_ and α_v_β_5_, which are also upregulated in HCC cells (Xiao et al., 2021). The POSTN-integrin interaction is central to HCC progression, influencing several downstream signaling pathways that support tumor development (Xiao et al., 2021; Wang et al., 2024a).

**FIGURE 2:**
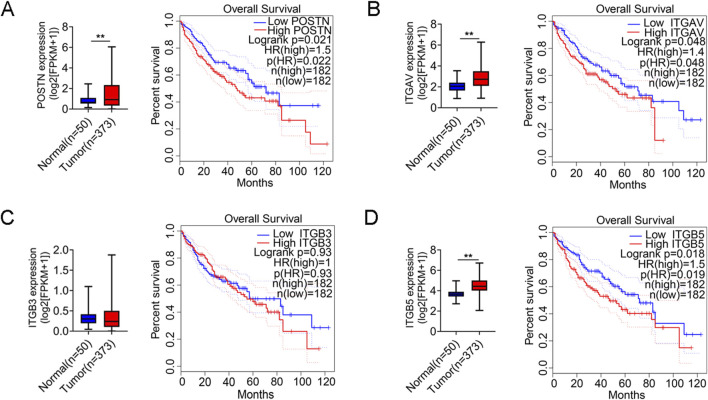
POSTN and integrin (α_v_β_3_ and α_v_β_5_) are highly expressed in tumor tissues of HCC patients. Differential expression analysis of **(A)** POSTN, **(B)** ITGAV, **(C)** ITGB3 and **(D)** ITGB5 between normal and tumor tissues in TCGA-LIHC. The LIHC tumor samples in the TCGA database were divided into high and low groups according to the median mRNA expression, and Kaplan-Meier survival analysis was performed. Student’s t-test was used for variable differences between the two groups. **p < 0.01, LIHC: Liver Hepatocellular Carcinoma.

To validate these findings, we analyzed POSTN mRNA expression using a transcriptomic dataset of 373 HCC tumors and 50 normal tissues from the Cancer Genome Atlas (TCGA) project. POSTN is significantly elevated in tumor tissues compared to normal tissues, and high expression levels of POSTN are associated with poorer overall survival (Figure 2A). In addition, differential expression and survival analysis of POSTN-bound integrins (α_v_β_3_ and α_v_β_5_) in HCC were also performed. The expression levels of integrin subunit alpha V (ITGAV) and integrin subunit beta 5 (ITGB5) were significantly increased in tumor tissues and correlated with poor prognosis of HCC patients (Figures 2B,D). The expression level of integrin subunit beta 3 (ITGB3) tends to increase in tumor tissues, and HCC patients with high expression of ITGB3 also have poor overall survival (no statistical significance), which may be related to the limited sample size of the TCGA database.

## 4 HSCs and POSTN^+^ CAFs

The malignant pre-microenvironment of HCC is seeded by liver injury, viral infection, inflammation and fibrosis, often accompanied by activation of quiescent mesenchymal cells into collagen-producing CAFs. CAFs promote immune escape, tumor metastasis, and drug resistance by remodeling the ECM and secreting growth factors and cytokines (Liu Y. et al., 2024; De Jaeghere et al., 2019; Li et al., 2021; Sun et al., 2023). CAFs affecting cancer progression and immune regulation are highly heterogeneous, with two distinct functions: pro-cancer CAFs and anti-cancer CAFs. Pro-cancer CAFs coordinate ECM remodeling and promote tumor inflammation, and regulate the immune microenvironment leading to immunosuppression, such as myofibroblast CAFs (myCAFs) and inflammatory CAFs (iCAFs) (Monteran and Erez, 2019). Conversely, anti-cancer CAFs are able to stimulate T cell activation associated with major histocompatibility complex (MHC) Class II expression, thereby improving immunotherapy strategies such as antigen-presenting CAFs (apCAFs) (Friedman et al., 2020). Quiescent HSCs have been identified as the major source of activated collagen-derived HSCs and CAFs in HCC and liver metastases using single-cell transcriptomics (Cogliati et al., 2023). POSTN^+^ CAFs have recently been reported to differentiate from HSCs (Ramachandran et al., 2019), predominantly located in the peripheral tumor region and dominates ECM remodeling compared to other CAF subsets (Wang et al., 2024a; Li Z. et al., 2024). POSTN^+^ CAFs exhibit characteristics of myCAFs and iCAFs due to their expression of inflammatory signature genes (C-X-C motif chemokine ligands (CXCL9/10/11)) and ECM-related signature genes (POSTN, metalloproteinase (MMP11/14)) (Wang et al., 2024a). Notably, POSTN^+^ CAFs specifically expressed the endoglin gene (encoding CD105), similar to CD105-positive tumorigenic pancreatic fibroblasts (Li Z. et al., 2024). HCC patients with more POSTN^+^ CAFs had shorter overall survival and progression-free survival, indicating that POSTN^+^ CAFs may promote tumor progression and adversely affect patient prognosis (Wang et al., 2024a).

**FIGURE 3:**
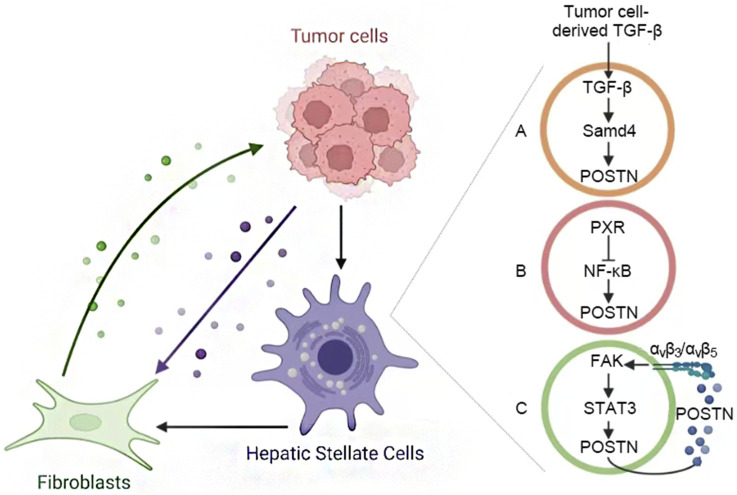
Activation of POSTN^+^ CAFs. **(A)** Tumor TGF-β induces the expression of POSTN in HSCs, POSTN activating HSC promotes the expression of TGF-β in HCC cells. **(B)** NF-κB regulates POSTN expression. The activation of PXR in HSC can inhibit this process and prevent HSC activation. **(C)** The POSTN of HSC binds to integrins and forms an autocrine positive feedback through the FAK/STAT3 pathway to promote its activation.

POSTN^+^ CAFs can promote angiogenesis and inhibit immune response on HCC cells by activating ECM, hypoxia and TGF-β signaling pathways. In addition, tumor cells can also promote HSC activation into CAF (including POSTN^+^ CAFs), and the bidirectional interaction between them forms a positive feedback loop to promote tumor growth, invasion and metastasis. The mechanism of POSTN mediated HSC activation in HCC TME mainly includes three pathways (Figure 3): (A) Tumor cell-derived TGF-β induces POSTN expression in HSCs via the suppressor of mother against decapentaplegic (SMAD) pathway (Liu B. et al., 2024). In turn, activated HSC-derived POSTN increases the expression of TGF-β by binding to HCC cell surface integrins. (B) The nuclear factor κB (NF-κB) directly regulates POSTN expression, while pregnane X receptor (PXR) activation in HSCs inhibits NF-κB-mediated POSTN transcription, thereby inhibiting HSC activation (Sato et al., 2024). (C) HSC-derived POSTN binds with integrins α_v_β_3_ and α_v_β_5_ to promote HSC activation through focal adhesion kinase (FAK)/signal transducer and activator of transcription 3 (STAT3)/POSTN signaling pathway, forming an autocrine positive feedback signal loop [9, 36].

## 5 Biological function of POSTN-integrin signaling in HCC progression

**FIGURE 4:**
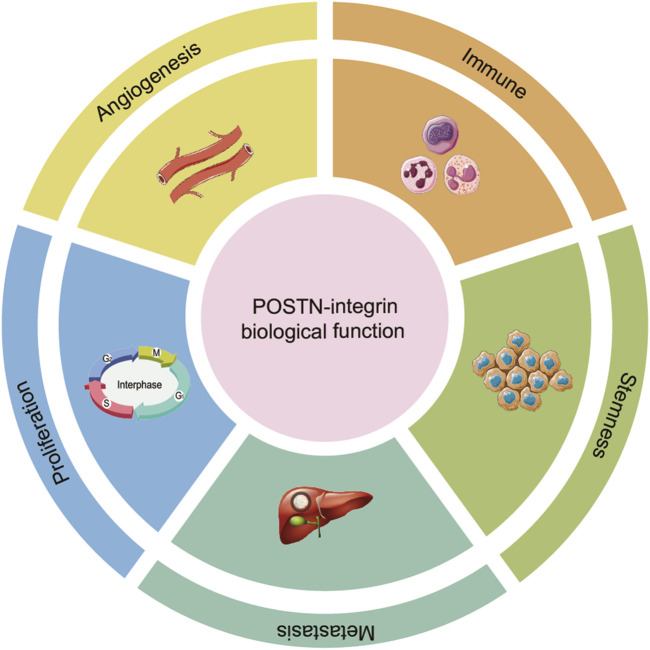
The role of POSTN-integrin signaling in the progression of HCC. The binding of POSTN to integrins initiates a downstream signaling cascade reaction, influencing a wide range of cellular processes, including proliferation, metastasis, immune escape, stemness and angiogenesis.

POSTN interacts with integrin receptors, particularly α_v_β_3_ and α_v_β_5_, establishing a critical signaling nexus that enhances tumor cell behavior in HCC progression. The binding of POSTN to these integrins initiates downstream signaling cascades that is a key driver of HCC progression, influencing a wide range of cellular processes, including tumor cell proliferation, metastasis, immune escape, cancer stemness and angiogenesis (Figure 4).

### 5.1 Tumor cell proliferation

Tumor cells usually acquire the ability to proliferate continuously. Normal tissues can precisely control the generation and release of growth-promoting signals, which can guide the initiation and progression of the cell proliferation-differentiation cycle, thus maintaining the balance of cell number and ensuring the structure and function of normal tissues (Parker et al., 2020; Hanahan, 2022). Cancer cells interfere with these signals and autonomously control cell growth. There are two possible ways in which POSTN promotes HCC cell proliferation: On the one hand, POSTN produced by cancer cells directly acts on themselves through autocrine to promote proliferation. POSTN mRNA expression was positively correlated in tumor tissues, but not in non-tumor tissues. POSTN knockout of HCC cells inhibited cell proliferation both *in vivo* and *in vitro* (Wu et al., 2022). The upregulated POSTN protein of HCC cells bind to the integrin α_v_β_3_ receptor on the cell surface membrane, promoting the expression and release of TGF-β1. TGF-β1 binds to receptors on the cell membrane and promotes the expression and secretion of POSTN protein, forming a positive feedback loop between POSTN and TGF-β1 (Chen et al., 2021). On the other hand, POSTN protein derived from stromal cells in the TME activates the proliferation signal of HCC cells by paracrine binding to integrins on the surface of the cell membrane. HSC-derived POSTN significantly enhanced the proliferation ability of heat-exposed residual HCC cells (Zhang R. et al., 2018). In addition, Xiao et al. showed that HSC-derived POSTN activates extracellular signal-regulated kinase (ERK) signaling pathways in HCC cells in a paracrine manner, leading to increased cell proliferation, and this signal transduction mechanism contributes to the development of HCC (Xiao et al., 2021).

### 5.2 Angiogenesis

The growth and local invasion of HCC is achieved through the formation of new blood vessels (Yao et al., 2023). The expansion of tumor mass stimulates angiogenesis, and the tumor mass secretes pro-angiogenic factors, which then induces the activation and proliferation of endothelial cells in a paracrine manner, so that the original blood vessels germinate new blood vessels. Angiogenesis is a process regulated by several growth factors, including vascular endothelial growth factor (VEGF), platelet-derived growth factor (PDGF), and basic fibroblast growth factor (FGF) (Vimalraj, 2022; Hosaka et al., 2020; Lugano et al., 2020). POSTN has been shown to promote angiogenesis by enhancing the angiogenic ability of endothelial cells. Previous studies have found that the expression level of POSTN is positively correlated with the expression level of VEGF in HCC (Lv et al., 2013a). In addition, Chen et al. found that sulfatase-2 promotes the sustained production of angiogenic factors through TGF-β/SMAD-dependent upregulation of POSTN, thereby promoting HCC angiogenesis (Chen et al., 2017). A technical barrier to anti-angiogenic therapy is the emergence of resistance, as a hypoxic response often occurs within the tumor, triggering a cascade of changes. Wang et al. found that POSTN improved the survival rate of tumor cells and promoted tumor angiogenesis under hypoxia conditions (Wang W. et al., 2011). Therefore, anti-angiogenic therapy combined with POSTN-targeted therapy may be an effective strategy.

### 5.3 Metastasis

The degree of malignancy and aggressiveness can be reflected by the degree of metastasis and invasion of the tumor, metastasis remains the leading cause of HCC-related death (Niu et al., 2022; Zhou et al., 2023). Despite significant advances in the combined treatment of HCC, long-term survival remains limited due to high rates of postoperative recurrence and metastasis. Approximately 60%–70% of HCC patients have recurrence or metastasis within 5 years after radical resection (Llovet et al., 2021). POSTN plays a key role in promoting angiogenesis and pre-metastasis of various cancers (Wasik et al., 2022). HCC is a highly vascularized tumor, and most HCC metastases are formed through the vasculature. Remodeling of blood vessels is essential to support progression and promote local and distant invasion of tumors (Liu et al., 2023). POSTN secreted by HCC cells leads endothelial cells to activate POSTN downstream signals through phosphorylation of FAK and phosphorylation of protein kinase B (AKT), which promotes HCC angiogenesis and thus metastasis (Chen et al., 2017). Bone marrow-derived endothelial progenitor cells (EPCs) have been identified as key angiogenic switches for metastasis in addition to endothelial cells (Shih et al., 2015). These subpopulations have the ability to integrate into tissues and form vascular structures that support cancer angiogenesis, and further differentiate into endothelial cell lines with strong colony-forming capabilities (Viallard and Larrivée, 2017). Hepatogenic POSTN upregulates C-C motif ligand 2 (CCL2) expression in EPCs via α_v_β_3_/integrin-linked kinase (ILK)/NF-κB pathway, while CCL2 further induces CD36 expression via CC chemokine receptor 2 (CCR2)/STAT3 pathway by directly binding to the CD36 promoter region, thereby promoting the premetastatic properties of HCC (Deng et al., 2024).

POSTN is secreted into the extracellular stroma to form ECM, creating a favorable microenvironment for tumor cell migration and invasion. miR-876 and neurogenic locus notch homolog (Notch) signaling directly regulate POSTN transcription in HCC cell lines, which could affect ECM organization and facilitate the transformation and invasion of tumor cells (Chen B. C. et al., 2020; Kongkavitoon et al., 2018). Activated HSCs are important mesenchymal cells in the HCC microenvironment. POSTN promotes the activation of HSCs to promote EMT of HCC cells (Sato et al., 2024). In addition, POSTN secreted by activated HSCs promoted the metastasis of heat-exposed residual HCC cells (Zhang Z. L. et al., 2018).

### 5.4 Immune escape

Recent advances in tumor immunotherapy have highlighted the importance of TME. The biological functions of POSTN on immune cells are firstly derived from the combination of POSTN with integrins on the surface of immune cells to regulate downstream signal transduction; secondly, POSTN mediates the physical and molecular properties of ECM to regulate immune cell localization and migration.

#### 5.4.1 Macrophage

Tumor-associated macrophages (TAM) are one of the most abundant immune cells infiltrating TME and present in all stages of HCC progression (Cheng et al., 2022; Ruf et al., 2023; Sung, 2022; Lu et al., 2023). Targeting TAM has become one of the most popular immunotherapy strategies. TAM is highly heterogeneous and performs different functional switches according to the TME, differentiating into classical active type (M1 phenotype) and replacement active type (M2 phenotype) (Gao et al., 2022; Nasir et al., 2023; Toledo et al., 2024). CAFs promote monocyte recruitment and differentiation into M2 macrophages through a variety of secreted molecules, including POSTN, which in turn impair effector T cell responses and induce an immunosuppressive microenvironment in HCC. Osteopontin (SPP1)^+^ macrophages were found to be more prevalent in anti-inflammatory responses and skewed towards the M2 phenotype (Glaviano et al., 2023; Coulis et al., 2023). Recently, SPP1^+^ macrophages were found to be the cell subtype most associated with POSTN^+^ CAFs interaction and associated with poor overall survival in HCC patients (Wang et al., 2024a). Mechanistically, POSTN^+^ CAFs promote the overexpression of SPP1 in macrophages through the IL-6/STAT3 signaling pathway, leading to the differentiation of macrophages into the SPP1^+^ phenotype (Wang et al., 2024a). Another study used spatial transcriptomic analysis to reveal crosstalk and co-localization between POSTN^+^ CAF, folate receptor 2 (FOLR2)^+^ macrophages and plasma-vesicular associated protein (PLVAP)^+^ endothelial cells (Li Z. et al., 2024). The high expression of fibroblast activation protein (FAP) in CAFs interacts with POSTN gene to increase the invasion of THP-1 cells and induce them to polarize into M2 macrophages (Cai et al., 2023a).

#### 5.4.2 Neutrophil

The importance of gut microbiota in regulating systemic immunity has been widely recognized. Microbiota imbalance exists in different stages of chronic liver disease, which inhibits immune monitoring. Trimethylamine (TMA) is first formed by initial catabolic intestinal microorganisms and then efficiently metabolized by flavin monooxygenase family enzymes to form Trimethylamine N-oxide (TMAO) (Wang Z. et al., 2011). TMAO upregulates POSTN and activates the ILK/AKT/mammalian target of rapamycin (mTOR) pathway in HCC, and the high expression level of POSTN is closely related to neutrophil infiltration of HCC patients (Wu et al., 2022).

#### 5.4.3 T cell

T lymphocytes are the key immune cells in the tumor immune microenvironment, among which CD8^+^ T cells, CD4^+^ T cells, and regulatory T lymphocytes (Treg) subsets have received extensive attention in the development and immunotherapy of HCC (Sangro et al., 2021; Du et al., 2024; Wang et al., 2024b). ECM remodeling is an important factor in tumor progression and generates a physical barrier that inhibits the recruitment of immune cells, especially T cells, to cancer sites (Zhang et al., 2025; Lu et al., 2025; He et al., 2021). The genes upregulated in POSTN^+^ CAFs were significantly enriched in several pathways related to ECM remodeling in the TME, including ECM receptor interaction, AKT/mTOR signaling pathway, focal adhesions and TGF-β signaling pathway (Wang et al., 2024a). POSTN can reshape the structure and composition of the ECM, alter the migration and infiltration abilities of T cells in the TME, making it difficult for T cells to reach the area where tumor cells are located, and thus unable to exert effective killing effects. In HCC patients, CD68 and Collagen type I α 1 (COL1A1) expression levels were increased in regions with high POSTN expression, whereas POSTN levels were lower in regions with high CD3D expression (Wang et al., 2024a; Li Z. et al., 2024; Li Y. et al., 2024). Furthermore, Numerous studies have highlighted the key role of ECM-associated CAFs in promoting immunotherapy resistance by promoting T cell exclusion (Kieffer et al., 2020; Jenkins et al., 2022; Zhu et al., 2023). When POSTN is abnormally high expressed in HCC TME, T cells can be induced to enter the exhaustion state. There was a significant negative correlation between POSTN^+^ CAFs infiltration and CD8^+^ T cell infiltration, while there was a significant positive correlation between POSTN^+^ CAFs and T cell exclusion, which is responsible for the exclusion of T cells from malignant regions in HCC tumor tissue (Wang et al., 2024a).

Meanwhile, POSTN also plays an important role in the CAFs-driven immune suppression process. POSTN can induce CAFs to express more immunosuppressive molecules, such as programmed death ligand 1 (PD-L1), which strongly inhibit the activation and function of T cells after binding to the corresponding receptors on the surface of T cells. For example, patients with a high proportion of POSTN^+^ CAFs have significantly worse responses to PD-1 treatment than other patients (Wang et al., 2024a). In addition, based on the anti-PD-1 treatment experiment of the orthotopic liver cancer implant model, compared with the adeno-associated viruse (AAV)-control group and the IgG combination treatment group, the AAV-shPOSTN and IgG combination treatment group significantly inhibited tumor proliferation, further verifying that POSTN knockdown can promote PD-1 monoclonal antibody treatment *in vivo* (Wang et al., 2024a).

### 5.5 Cancer stemness

Cancer stem cells (CSCs) play an important role in the metastasis, recurrence and chemoresistance of HCC. Since the concept of CSCs was proposed, its origin has been controversial. Most researchers believe that CSCs in many tumors (including HCC) are derived from the abnormal proliferation and differentiation of normal stem cells. Other studies have suggested that differentiated mature tumor cells can also generate CSCs in response to inducing factors (Zeng et al., 2023). The components in the TME play an important role in the process of transforming tumor cells into CSCs. Song et al. found that CD133^+^ HCC cells have stronger invasion, metastasis and tumorigenesis capabilities, and proposed that CD133 is a marker of CSCs. POSTN in the TME promotes the direct binding of the transcription factor adaptor protein complex 2α (AP-2α) to the CD133 promoter region, thereby enhancing the expression of CD133 gene in HCC cells (Chen et al., 2021).

POSTN enhances the tolerance of CSCs to conventional chemotherapy. Zhang et al. found that POSTN secreted by activated HSCs induced residual HCC cells to acquire stem-like properties after incomplete thermal ablation (Zhang et al., 2017). The molecular mechanism is that POSTN regulates the stemness of heat-exposed residual HCC cells through integrin β1/AKT/glycogen synthase kinase-3β (GSK-3β)/β-catenin/transcription factor 4 (TCF4)/Nanog signaling pathway (Zhang et al., 2017). CSCs refill the tumor after initial treatment, resulting in poor response to radiotherapy and chemotherapy in HCC patients. Therefore, future HCC therapies could focus on targeting CSCs rather than simply killing HCC cells.

## 6 Molecular mechanism of POSTN-integrin regulating HCC progression

**FIGURE 5:**
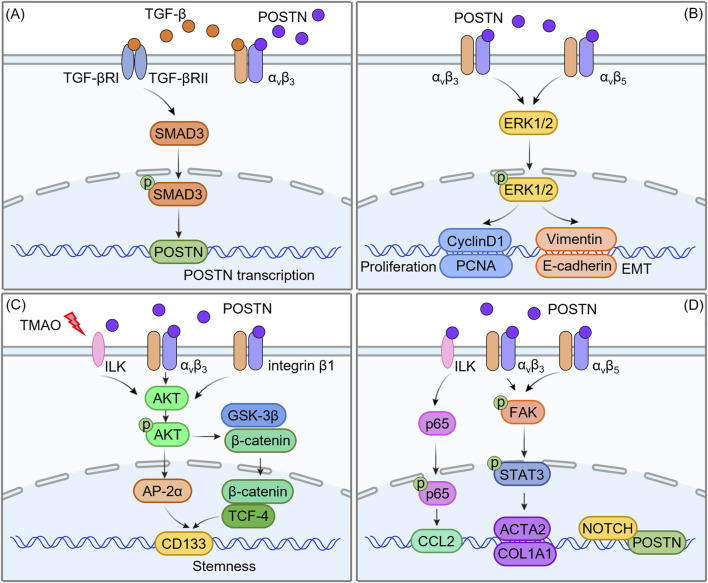
Molecular mechanism of POSTN regulating HCC progression. **(A)** The positive feedback loop of POSTN-integrin and TGF-β/SMAD signaling pathway. **(B)** Cross-regulation of POSTN-integrin with the MAPK/ERK signaling pathway. **(C)** The interaction between POSTN-integrin and the AKT/mTOR signaling pathway. **(D)** POSTN-integrin and other pathways.

POSTN binds integrins to interact with various intracellular signaling pathways, which is one of its core biological functions. Numerous evidences have shown that POSTN can promote the progression of HCC in a synergistic manner with TGF-β/SAMD signaling pathway, MAPK/ERK signaling pathway and AKT/mTOR signaling pathway. Furthermore, POSTN can form complex crosstalk with other pathways in an upstream and downstream relationship. This article will elucidate the molecular mechanism by which POSTN regulates HCC progression (Figure 5; Table 1).

**TABLE 1:** The molecular mechanism of POSTN-integrin regulating the progression of HCC.

Signaling pathway	The interaction between POSTN and signal pathways	References
TGF-β/SMAD signaling pathway	TGF-β/SMAD signaling upregulates the expression of POSTN	Chen et al. (2017), Chen et al. (2021)
	POSTN promotes the release of active TGF-β1 by activating integrin α_v_β_3_	Chen et al. (2021)
MAPK/ERK signaling pathway	POSTN promotes the phosphorylation of ERK1/2 to regulate tumor cell cycle and proliferation	Xiao et al. (2021)
	POSTN-integrin-MAPK/ERK axis enhances the metastatic potential of HCC cells	Chen et al. (2019)
AKT/mTOR signaling pathway	POSTN-initiated AKT/mTOR activation enhances the stemness of HCC cells	Chen et al. (2021), Zhang et al. (2017)
	POSTN promotes the proliferation of HCC cells through the AKT/mTOR pathway	Wu et al. (2022)
FAK/STAT3 signaling pathway	POSTN activates the FAK/STAT3 signaling pathway to promote HSC activation	Xiao et al. (2021)
Notch signaling pathway	Notch mediates POSTN transcriptional regulation to promote tumor cell proliferation	Kongkavitoon et al. (2018)
NF-κB signaling pathway	POSTN promotes premetastatic properties of HCC through the α_v_β_3_/ILK/NF-κB pathway	Deng et al. (2024)

### 6.1 The positive feedback loop of POSTN-integrin and TGF-β/SMAD signaling pathway

TGF-β is a multifunctional cytokine with multiple roles in various physiological and pathological processes. TGF-β signaling is initiated by binding of TGF-β to two types of cell surface receptors: TGF-β type I receptor (TGF-βRI) and TGF-β type II receptor (TGF-βRII) (Ren et al., 2022; Liu et al., 2022; Lind et al., 2020). TGF-βRII, as a high-affinity TGF-β receptor, recruits and phosphorylates the intracellular domain of TGF-βRI to form a heterotetramer, thereby activating the downstream signaling SMAD protein (Aashaq et al., 2022). TGF-β induces apoptosis (programmed cell death) and inhibits cell proliferation by activating the pro-apoptotic protein bax in the early stage of tumor development. However, TGF-β promotes the progression of advanced tumors. This pro-tumor effect is attributed to its ability to regulate multiple cellular processes such as genomic instability, EMT and immune escape (Glaviano et al., 2023; Peng et al., 2022; Shi et al., 2022).

In recent years, increasing evidence has demonstrated a synergistic effect between POSTN-integrin and TGF-β/SMAD signaling pathway, which is particularly significant in the remodeling of HCC TME. On the one hand, TGF-β1 upregulates the expression of POSTN by activating the SMAD3 signaling axis (Chen et al., 2017): TGF-β1 binds to the receptor and initiates signal transduction, resulting in intracellular SMAD3 phosphorylation modification. As a functional transcription factor, phosphorylated SMAD3 can directly recognize and bind to specific response elements in the promoter region of POSTN gene, thereby initiating the transcription and expression of POSTN and promoting its secretion in the ECM. On the other hand, POSTN in turn can enhance the function of TGF-β/SMAD signaling pathway by regulating the active release of TGF-β (Chen et al., 2021): TGF-β mainly exists in the “latent form” in the ECM. It forms a complex with latent TGF-β binding protein (LTBP) and is anchored in the ECM through LTBP. POSTN, as a secreted ECM protein, specifically binds to the integrin α_v_β_3_ receptor on the cell surface and activates downstream signaling cascades to trigger the release of latent TGF-β1 from the α_v_β_3_/LTBP complex and conversion into a biologically active form.

This interaction eventually forms an efficient “POSTN-integrin-TGF-β/SMAD positive feedback loop”: TGF-β upregulates the expression of POSTN by activating SMAD3, and POSTN promotes the release of active TGF-β1 by activating integrin α_v_β_3_. The released TGF-β1 further activates the SMAD3 signal, and this cycle repeats itself, continuously amplifying the synergistic effect of the two. This loop in HCC TME can significantly enhance the proliferation, migration and invasion ability of tumor cells, and promote angiogenesis and matrix remodeling, which becomes an important molecular mechanism to promote tumor progression and metastasis.

### 6.2 Cross-regulation of POSTN-integrin with the MAPK/ERK signaling pathway

Mitogen-activated protein kinases (MAPK) signaling pathway is an important signaling pathway that transmits extracellular signals such as cytokines, hormones and cellular stress into the cell, and its dysfunction is closely related to abnormal activation and poor prognosis of various cancers (Bahar et al., 2023; Huang et al., 2023). ERK plays a key role in signal transduction as an affecting kinase in the upstream signal of MAPK signaling pathway (Crozet and Levayer, 2023). The MAPK/ERK pathway is another frequently abnormally activated signaling pathway in HCC, and the activation of this pathway usually begins with the binding of cell surface receptors, such as growth factor receptors, to ligands, activation of ERK1/2 by phosphorylation via the RAS/RAF/MAP kinase-ERK kinase (MEK)/ERK cascade, and then to the nucleus to regulate the transcription of target genes (Roberts and Der, 2007). It is worth noting that recent studies have identified POSTN as a key regulator of the MAPK/ERK pathway in HCC. Clinical evidence from immunohistochemical analysis of HCC tissues indicates that there is a strong positive correlation between POSTN overexpression and elevated phosphorylated ERK1/2 (p-ERK1/2) levels (Chen et al., 2019). Furthermore, patients co-expressing high levels of POSTN and p-ERK1/2 exhibited a more aggressive tumor phenotype (Chen et al., 2019): Compared with patients with low expression of these two molecules, they showed greater depth of tumor invasion, higher rates of lymph node metastasis, and more distant metastases. Importantly, these patients also demonstrated poor overall survival, highlighting the clinical relevance of this correlation.

Mechanistic studies have elucidated that the POSTN-integrin axis is a key upstream regulator of the MAPK/ERK signaling pathway in HCC. POSTN binds integrin α_v_β_3_ and α_v_β_5_ with high affinity, triggering conformational changes in the integrin complex and activating downstream signaling molecules. A key downstream effect is FAK, a non-receptor tyrosine kinase that localizes to focal adhesion, and activated FAK then initiates a signaling cascade that converges on the RAS/RAF/MEK/ERK axis: POSTN binds to integrin (α_v_β_3_/α_v_β_5_) and then phosphorylates ERK1/2 by activating FAK. The activated ERK1/2 enters the nucleus and continuously activates Cyclin D1 and proliferating cell nuclear antigen (PCNA), disrupting the balance of cell cycle regulation and leading to abnormal proliferation of hepatoblastoma cells (Xiao et al., 2021). In addition to promoting cell proliferation, the POSTN-integrin-MAPK/ERK axis enhances the metastatic potential of HCC cells. Mechanistically, POSTN inhibits E-cadherin transcription and upregulates vimentin expression by promoting ERK1/2 phosphorylation (Chen et al., 2019). The loss of E-cadherin and the gain of vimentin enable HCC cells to escape from the primary tumor mass and degrade the surrounding ECM through matrix metalloproteinases (MMPs). This drives the EMT process to acquire a mesenchymal phenotype and migrate through the blood or lymphatic system to form distant metastases.

Together, these findings highlight the critical role of cross-regulation between the POSTN-integrin and the MAPK/ERK signaling pathway in HCC pathogenesis. This crosstalk not only promotes tumor cell proliferation by disrupting cell cycle homeostasis, but also enhances tumor invasion and metastasis through EMT induction. Combined targeting of POSTN-integrin and MAPK/ERK signaling may play a synergistic role in inhibiting HCC growth and metastasis, as it simultaneously blocks upstream ECM-initiated signaling and downstream kinase cascades.

### 6.3 The interaction between POSTN-integrin and the AKT/mTOR signaling pathway

The AKT/mTOR pathway is an important signal transduction pathway within cells, involved in regulating various biological processes such as cell proliferation, survival, metabolism, and angiogenesis (Yu et al., 2022; Tian et al., 2023). As a core executive molecule in this pathway, AKT triggers a cascade of downstream events that converge on cell growth and proliferation through phosphorylation activation at key residues (Thr308 and Ser473) (Yu et al., 2022). Dysregulation of AKT/mTOR signaling is frequently observed in the context of HCC, and the integrin-mediated interaction between POSTN and AKT/mTOR pathway becomes a key regulatory axis for HCC progression.

POSTN, when bound to integrin (α_v_β_3_), initiates downstream signal transduction by promoting AKT phosphorylation. Activated AKT directly phosphorylates mTOR and activates its downstream effector molecule AP-2α, a transcription factor that drives the expression of a CSC-related molecule (CD133), which is essential for maintaining the self-renewal ability and treatment resistance of HCC cells. It emphasized how POSTN-initiated AKT/mTOR activation enhances the stemness of HCC cells (Chen et al., 2021). In addition to the typical α_v_β_3_ integrin, the regulation of HCC stemness by POSTN extends to other integrin subtypes under specific pathological conditions. For example, POSTN binds to integrin β1 in heat-exposed residual HCC cells, triggering a unique but interrelated signaling cascade: activated integrin β1 promotes AKT phosphorylation, which in turn phosphorylates GSK-3β. Phosphorylation deactivates GSK-3β and prevents it from degrading β-catenin. Stable β-catenin subsequently translocate to the nucleus where it forms a complex with TCF4 to induce the expression of CSC-related genes (Zhang et al., 2017). This mechanism not only explains how POSTN maintains the stemness of residual HCC cells after heat-exposed, but also highlights its environment dependent role in activating AKT-mediated pathways.

The emerging evidence further links the regulation of POSTN to environmental factors, especially the gut-liver axis. Zhao et al. demonstrated that TMAO, a metabolite produced by the gut microbiota, upregulates the expression of POSTN in HCC cells (Wu et al., 2022). Functionally, the TMAO-POSTN axis amplifies AKT/mTOR signaling (Wu et al., 2022): knockdown of POSTN by RNA interference in HCC cell lines (such as Hepa1-6 and Huh7) resulted in significant reductions in AKT phosphorylation and mTOR activity, accompanied by impaired cell proliferation. These findings suggest that POSTN is a key upstream regulator of AKT/mTOR signaling in HCC, linking microbial metabolism to tumor progression.

Collectively, the accumulated evidence highlights POSTN as a nodal molecule that links extracellular signals such as ECM interactions, microbial metabolites to the intracellular AKT/mTOR pathway to drive HCC genesis, proliferation, and treatment resistance. In view of this, jointly targeting the POSTN-integrin and the AKT/mTOR signaling pathway can synergically disrupt the tumor-promoting signaling network. This combination strategy can overcome the limitations of monotherapy and provide new treatment approaches for HCC patients, especially those with advanced or recurrent diseases.

### 6.4 POSTN-integrin and other pathways

In addition to the above three key signaling pathways, POSTN can also regulate the biological processes of HCC through other complex signaling networks. Specifically, POSTN was found to promote the expression of smooth muscle α-actin 2 (ACAT2) and COL1A1 by activating FAK/STAT3 signaling in HCC TME (Xiao et al., 2021). This cascade holds significant biological importance: FAK is a key mediator for cell adhesion and cytoskeletal remodeling, and its activation can trigger downstream STAT3 phosphorylation. Phosphorylated STAT3 is then transported to the nucleus, where it binds to the promoter regions of ACAT2 and COL1A1, enhancing their transcription. It is worth noting that the upregulation of ACAT2 (a key molecule for smooth muscle cell differentiation) and COL1A1 (a major component of the ECM) further promotes the activation of HSCs. POSTN secreted by activated HSCs forms a positive feedback loop in an autocrine manner, leading to sustained HSCs activation, and also participates in the construction of TME in a paracrine manner, accelerating the progression of HCC (Xiao et al., 2021).

In the context of POSTN autotranscriptional regulation, immunoglobulin κJ-region recombinant signal binding protein (RBPJ), a DNA-binding protein that serves as a core partner of the Notch1 receptor, has been identified as a key regulator. There are five RBPJ binding regions in the −5,000/+5,000 bp range relative to POSTN transcription start sites, a broad genomic window suggesting that RBPJ may fine-tune POSTN expression (Kongkavitoon et al., 2018). Notch1 associates with these RBPJ binding sites after activation in liver bile duct carcinoma cells, and this interaction may promote Notch1-mediated regulation of POSTN transcription, as the Notch1/RBPJ complex is known to control cell fate decisions, proliferation, and differentiation in cancer (Kongkavitoon et al., 2018).

Furthermore, the research by Deng et al. revealed another layer of POSTN-mediated signal transduction: POSTN derived from hepatocytes can upregulate the expression of CCL2 in EPCs through the α_v_β_3_/ILK/NF-κB pathway (Viallard and Larrivée, 2017). Mechanically, POSTN binds to the α_v_β_3_ integrin receptor on the surface of EPCs, initiating conformational changes in the receptor and activating ILK. Activated ILK then phosphorylates downstream targets, leading to nuclear translocation of NF-κB, which is a major regulator of inflammation and immune responses. NF-κB then binds to the CCL2 promoter, driving its transcription. Given that CCL2 is an effective chemical attractant for monocytes and macrophages, and EPCs are involved in vascular repair and angiogenesis, this pathway may play a key role in liver inflammation, angiogenesis or tissue repair, linking hepatocellular derived POSTN with paracrine regulation of EPC function.

## 7 Potential clinical applications of POSTN-integrin signaling

The increasing understanding of the molecular mechanisms underlying HCC progression has opened avenues for targeted therapeutic strategies. Among the various players in HCC, POSTN has garnered attention for its multifaceted role in tumor biology, particularly its involvement in the TME and signaling pathways that promote tumor growth, metastasis, and treatment resistance. Given its significant role in HCC pathogenesis, POSTN presents several potential clinical applications, ranging from diagnostic biomarkers to therapeutic targets.

### 7.1 POSTN as a marker for early diagnosis and prognosis of HCC patients

Abnormal POSTN expression in HCC patients is associated with pathological diagnosis. Studies have shown that overexpression of POSTN is associated with tumor aggressiveness, late metastasis and poor prognosis. POSTN is easily detected by blood, urine, interstitial fluid and other body fluids, so is expected to be a potential molecular marker for the early diagnosis of HCC patients (Table 2).

**TABLE 2:** Correlation between POSTN expression levels and clinicopathological parameters in HCC patients.

High expression in	Is associated with	References
Tumor tissues	Overall survival	Chen et al. (2017)
Tumor tissues	Tumor nodules, microvascular invasion, edmodson grade, TNM stage and overall survival	Lv et al. (2013a)
Tumor tissues	HBV, tumor grade and overall survival	Riener et al. (2010)
Tumor tissues	Microvascular invasion, tumor stage and overall survival	Jang et al. (2016)
Exosomal	HBV, exosome formation and adhesion	Todorova et al. (2023)
Serum	Edmodson grade and overall survival	Lv et al. (2013b)

#### 7.1.1 Tumor POSTN

POSTN was highly expressed in epithelial cells and tumor stroma of HCC. High expression of POSTN is associated with tumor nodules, microvascular invasion, edmodson grade, and tumor-lymph node-metastasis (TNM) stage, affecting the prognosis of patients and decreasing overall survival (Chen et al., 2017; Lv et al., 2013a; Riener et al., 2010; Jang et al., 2016). Exosomal POSTN is associated with viral activity, exosome formation and cell adhesion (Todorova et al., 2023). Through multivariate analysis, yang et al. found that POSTN overexpression could be used as a biomarker of cancer prognosis (Lv et al., 2013a). It has also been shown that POSTN can predict prognosis together with other factors. For example, the combination of the expression level of POSTN and the degree of microvascular invasion can better predict the prognosis of HCC patients (Jang et al., 2016). In addition, chen et al. have shown that POSTN and AP-2α play a role in HCC progression and patient survival (Chen et al., 2021).

#### 7.1.2 Serum POSTN

POSTN was significantly elevated in serum of HCC patients. Studies have shown that elevated serum POSTN is considered an independent prognostic indicator of survival (Lv et al., 2013b). Fujimoto et al. found that serum POSTN levels in patients with intrahepatic cholangiocarcinoma were significantly higher than those in patients with chronic progressive liver disease and HCC, suggesting that serum POSTN levels can be used to distinguish cholangiocarcinoma from other liver malignancies (Fujimoto et al., 2011).

### 7.2 Therapeutic targeting of POSTN-integrin signaling

#### 7.2.1 The targeting strategy of POSTN-integrin signals

POSTN-integrin signaling has great potential as a therapeutic target for inhibiting HCC progression. Strategies targeting POSTN can be categorized into several approaches: a. Pharmacological agents that inhibit the expression or secretion of POSTN by tumor cells or stromal cells may reduce HCC progression. For example, Calcitriol (vitamin D analogue) inhibits the tumor-promoting effect of POSTN on heat-treated residual HCC (Zhang Z. L. et al., 2018). In addition, small molecule inhibitors targeting the signaling pathways that drive POSTN expression, such as curcumol (Jia et al., 2020), inhibits HSC activation, and its potential to downregulate POSTN in HCC can be investigated. b. Developing therapeutic agents that block the interaction between POSTN and integrins could effectively disrupt the signaling cascades that promote HCC cell survival, proliferation, and invasion. This could be achieved using peptides or inhibitors that specifically inhibit POSTN-integrin binding, such as peptides targeting CD51 or selective γ-secretase inhibitor LY3039478 (Cai et al., 2023b), thereby attenuating the downstream signaling pathways associated with tumor growth. c. Combining POSTN-targeted therapies with existing treatment modalities, such as chemotherapy, immunotherapy, or targeted therapies, may enhance therapeutic efficacy. For instance, POSTN contributes to drug resistance in HCC. It has been reported that POSTN is involved in the resistance of HCC cells to arsenic trioxide under hypoxia conditions (Liu et al., 2017). Soletinib combined with lenvatinib may be a promising therapeutic strategy for the treatment of HCC with high POSTN expression (Chen et al., 2021). In addition, in the postoperative prevention and treatment of HCC with high POSTN expression, the application of cilengitide combined with levastinib provides a feasible plan for clinical treatment (Chen et al., 2021). The combination of cilengitide and γ-secretase inhibitor LY3039478 can significantly inhibit the invasion and metastasis of HCC (Cai et al., 2023b). POSTN inhibition could sensitize HCC cells to chemotherapeutic agents by disrupting the protective TME, thereby enhancing drug delivery and efficacy.

#### 7.2.2 The main challenges of targeting POSTN-integrin signaling

Although POSTN is a promising therapeutic target, its complex biological characteristics pose multiple challenges to drug development. Firstly, POSTN has multiple splicing isomers, and the roles of different isomers in tumors may be completely different (Kudo, 2019). Most current treatment strategies are unable to distinguish these isomers, which may lead to the inhibition of beneficial POSTN functions (such as tissue repair), and fail to effectively block the cancer-promoting effect. For instance, POSTN short fragment with exon 17 promotes EMT and metastasis (Ikeda-Iwabu et al., 2021), while POSTN exon 21 antibodies inhibit tumor cell growth by reducing macrophage M2 polarization and the number of tumor blood vessels in triple-negative breast cancer (Fujikawa et al., 2021). Therefore, it is of vital importance to develop drugs that can specifically target cancer-promoting isomers. Precise targeting strategies based on CRISPR or RNA interference may be a promising direction, but problems such as delivery efficiency and off-target effects still need to be overcome.

Secondly, the sources of POSTN in the TME are heterogeneous. It can be secreted by tumor cells themselves or produced by CAFs. POSTN from different sources may play different roles. POSTN secreted by tumor cells mainly directly binds to integrins, activates the MAPK/ERK and AKT/mTOR signaling pathways, and thereby promotes metastasis and proliferation (Wu et al., 2022; Chen et al., 2019). On the other hand, the POSTN secreted by CAFs is more inclined to promote ECM remodeling, fibrosis and immunosuppression (Xiao et al., 2021; Wang et al., 2024a). This heterogeneity implies that simply targeting POSTN in tumor cells may not be sufficient to inhibit the CAF-driven tumor-promoting microenvironment. The ideal therapeutic strategy may require targeting both tumor cells and CAFs simultaneously, such as combining POSTN siRNA and FAP inhibitors.

Thirdly, tumors with high POSTN expression (such as HCC) usually have a dense fibrotic matrix (Chen K. et al., 2020), which constitutes the main obstacle to drug delivery. POSTN enhances the mechanical hardness of ECM by promoting collagen deposition and cross-linking, thereby restricting the penetration of antibodies, nanoparticles, and even immune cells. In addition, POSTN also increases interstitial hydraulics by regulating TGF-β signaling, further hindering the diffusion of drugs. These physical barriers limit the effectiveness of traditional chemotherapy and targeted therapy in tumors with high POSTN expression. To overcome this problem, researchers can explore combinations of matrix degraders (such as hyaluronidase) or develop new delivery systems (such as POSTN-reactive nanoparticles).

Finally, POSTN exerts its functions through multiple receptors and downstream signaling pathways, such as integrins, β-catenin and Notch (Kongkavitoon et al., 2018; Zhang et al., 2017). The redundancy of these signaling pathways may lead to drug resistance in targeted therapy. In addition, POSTN maintains the survival of CSCs by activating AKT/mTOR signaling (Chen et al., 2021), further increasing the difficulty of treatment. Future strategies to address this issue may require the development of multi-target inhibitors or the combination of epigenetic regulation to comprehensively block the adaptive response of tumors.

### 7.3 Implications for personalized medicine

The heterogeneity of HCC underscores the need for personalized treatment approaches. The characterization of POSTN expression profiles could lead to the stratification of patients into distinct molecular subtypes based on their POSTN levels and the associated signaling pathways. In clinical studies of HCC immunotherapy combination therapy, tumors of patients with higher levels of atezolizumab (anti-PD-L1) + bevacizumab (anti-VEGF) response signature genes showed more tumor-embryo reprogramming POSTN^+^ CAFs, TAMs, and endothelial cells, and the EMT-like features of cancer cells were also evident (Li Z. et al., 2024). This suggests that POSTN levels may influence the response of HCC patients to immunotherapy and thus the choice of treatment regimen. In the future, there may be great potential to start from POSTN^+^ CAFs, a group of “hub” cells, in terms of distinguishing the malignant degree of patients’ disease and developing innovative treatment strategies. This stratification may guide the selection of targeted therapies, improving treatment outcomes and minimizing unnecessary side effects.

## 8 Conclusion and future prospects

POSTN is a multifunctional ECM protein related to clinical diagnosis, patient prognosis, tumor cell proliferation and invasion, immune regulation and angiogenesis in HCC patients. Its molecular mechanism mainly involves TGF-β, ERK and AKT signaling pathway. There are still many questions about the role of POSTN in HCC that need further investigation. Since HCC is a complex disease, we should pay attention not only to the abnormal expression of POSTN inside tumor cells, but also to the POSTN protein secreted by CAFs. It is hoped that the systematic study of this research field will help us to have more understanding in the future.

Due to the heterogeneity of HCC cells and individual differences among HCC patients, it is difficult to find the best treatment strategy for each individual. Further understanding of the molecular mechanisms of HCC is expected to help us improve traditional therapies. Recent reports have shown that POSTN targeting in combination with other signaling inhibitors can achieve a sustained therapeutic response in HCC patients. In addition, the application of high-throughput sequencing technology in clinical patients will facilitate appropriate personalization of POSTN targets.

Future research should aim to clarify the precise roles of POSTN in various HCC subtypes and its interactions with other components of the TME. Investigating the signaling pathways activated by POSTN in different cellular contexts will provide insights into its multifaceted roles in HCC. Additionally, exploring the potential of POSTN as a therapeutic target in combination with immunotherapies may open new avenues for enhancing treatment responses.

Clinical trials are essential to evaluate the safety and efficacy of POSTN-targeted therapies and to determine their role in combination with existing treatments. Establishing POSTN levels as a predictive biomarker for response to therapy will further aid in the development of personalized treatment approaches.
